# Taxonomic and phylogenetic contributions to *Fuscoporia* (Hymenochaetales, Basidiomycota): two new species from Hawaii with a key to North American species

**DOI:** 10.3389/fcimb.2023.1205669

**Published:** 2023-06-21

**Authors:** Qian Chen, Lu Liu, Jing Si, Josef Vlasák

**Affiliations:** ^1^ College of Architecture and Urban Planning, Chongqing Jiaotong University, Chongqing, China; ^2^ College of Architecture and Urban Planning, Tongji University, Shanghai, China; ^3^ Institute of Microbiology, School of Ecology and Nature Conservation, Beijing Forestry University, Beijing, China; ^4^ Biology Centre, Institute of Plant Molecular Biology, Czech Academy of Sciences, Branišovská, České Budějovice, Czechia

**Keywords:** Hymenochaetaceae, phylogeny, polypore, taxonomy, new taxa

## Abstract

*Fuscoporia* is a cosmopolitan, poroid, wood-decaying genus, belonging to the Hymenochaetales. During a study of wood-inhabiting fungi in the USA, four unknown specimens were collected from Hawaii. Both morphological criteria and molecular genetic analyses based on the ITS+nLSU+EF1-α datasets and the nLSU dataset confirmed that these four specimens represent two new species of *Fuscoporia*, and they are described as *F. hawaiiana* and *F. minutissima. Fuscoporia hawaiiana* is characterized by pileate basidiocarps, the absence of cystidioles, hooked hymenial setae, broadly ellipsoid to subglobose basidiospores measuring 4−6 × 3.5−4.5 μm. *Fuscoporia minutissima* is distinguished by small pores (10−13 per mm) and basidiospores (3.4−4 × 2.4−3 μm). The taxonomic status of the two new species is briefly discussed. A key to the North American species of *Fuscoporia* is provided.

## 1 Introduction

Most wood-rotting fungi, belonging to Basidiomycetes, can use different types of wood as their nutritional source and are indispensable participants and important biological regeneration resources in natural ecosystems. The genus *Fuscoporia* Murrill (Hymenochaetales, Basidiomycota), erected by Murrill (1907) with *F. ferruginosa* (Schrad.) as generic type, is a cosmopolitan fungal group that encompasses also important species with medicinal effects, such as *F. gilva* (Schwein.) T. Wagner & M. Fisch. and *F. torulosa* (Pers.) T. Wagner & M. Fisch. (Wu et al., 2019). *Fuscoporia* is widely distributed in Asia, Europe, Oceania, and America. The genus has been considered a synonym of *Poria* Adans. or *Phellinus* Quél. for a long time (Overholts, 1953; Lowe, 1966; Ryvarden and Johansen, 1980; Gilbertson and Ryvarden, 1987; Larsen and Cobb-Poulle, 1990). However, Fiasson and Niemela (1984) recognized the genus as monophyletic morphologically, characterized by annual to perennial and resupinate to pileate basidiomata, a dimitic hyphal system with crystal encrustations on generative hyphae, the presence of hymenial setae, and hyaline, thin-walled and smooth basidiospores. Later, Wagner and Fischer, 2001; Wagner and Fischer, 2002) by means of nLSU sequence data and morphological studies of European Hymenochaetales reconfirmed the taxonomic status of the genus of *Fuscoporia*, with six species of *Phellinus* being classified into it.

Large number of *Fuscoporia* new species and new combinations have been discovered in recent years, and at present, more than 90 species are accepted in the genus (Chen et al., 2020; Tchoumi et al., 2020; Vlasák et al, 2020; Yuan et al., 2020; Dai et al., 2021; Chen et al., 2022; Hussain et al., 2022; Wu et al., 2022a, b). *Fuscoporia* is considered to be a complex genus with still unsettled taxonomy.

Striking diversity in the Hawaiian Islands with multiple co-occurring species leads to many species unreported in the literature. During a study of wood-inhabiting fungi, four unknown specimens were collected from Hawaii, with the features of *Fuscoporia*. To explore their relationships, phylogenetic analyses based on the ITS+nLSU+EF1-α datasets and the nLSU dataset were carried out. Both morphological criteria and molecular genetic analyses confirmed that these four specimens represent two new species of Fuscoporia. So, we describe them as *F. hawaiiana* and *F. minutissima* in the present paper. A key to the North American species of Fuscoporia is also provided.

## 2 Materials and methods

### 2.1 Morphological studies

Some studied specimen materials are deposited in the fungoria of the Institute of Microbiology, Beijing Forestry University (BJFC). The other ones are deposited in private fungoria of Josef Vlasak (JV) and then will be transferred into Prague Museum Herbarium (PRM). Morphological descriptions are based on field notes and herbarium specimens. The macroscopic color terms cited from Anonymous (1969) and Petersen (1996). Sections were studied at a magnification up to × 1,000 using a Nikon Eclipse 80i microscope with phase contrast illumination (Nikon, Tokyo, Japan). The microscopic analyses were performed accroding to Liu et al. (2022) and Si et al. (2023). Microscopic features, measurements, and drawings were prepared from slides stained with Cotton Blue. The following abbreviations are used: KOH = 5% potassium hydroxide, CB− = acyanophilous in Cotton Blue, IKI− = neither amyloid nor dextrinoid in Melzer’s reagent, L = arithmetic average of all spore length, W = arithmetic average of all spore width, Q = variation in the L/W ratios, and (n = x/y) = number of measured spores (x) measured from a given number of specimens (y).

### 2.2 DNA extraction, PCR, and sequencing

A CTAB rapid plant genome extraction kit (Aidlab Biotechnologies Co., Ltd., Beijing, China) was used to extract total genomic DNA from dried specimens following the manufacturer’s instructions with some modifications (Chen et al., 2020; Zhang et al., 2023; Zhou et al., 2023). For generation of PCR amplicons, the following primer pairs were used: ITS5 (GGA AGT AAA AGT CGT AAC AAG G) and ITS4 (TCC TCC GCT TAT TGATAT GC) for internal transcribed spacer (ITS, White et al., 1990); LR0R (ACC CGC TGA ACT TAA GC) and LR7 (TAC TAC CAC CAA GAT CT) for nuclear large subunit rDNA (nLSU, Vilgalys and Hester, 1990); EF1-983F (GCY CCY GGH CAY CGT GAY TTY AT) and EF1-1567R (ACH GTR CCR ATA CCA CCR ATC TT) for translation elongation factor-1 alpha (EF1-α, Rehner and Buckley, 2005). The PCR procedures for ITS and EF1-α were as follows: initial denaturation at 95°C for 3 min, followed by 35 cycles of denaturation at 94°C for 40 s, annealing at 54°C for 45 s and extension at 72°C for 1 min, and a final extension at 72°C for 10 min. The PCR procedure for nLSU was as follows: initial denaturation at 94°C for 1 min, followed by 35 cycles of denaturation at 94°C for 1 min, annealing at 50°C for 1 min and extension at 72°C for 1.5 min, and a final extension at 72°C for 10 min. The PCR products were purified and sequenced at the Beijing Genomics Institute with the same primers and the sequences are deposited in GenBank. All newly generated sequences were deposited in GenBank (http://www.ncbi.nlm.nih.gov ) and are listed in **Table 1**.

**Table 1:** Species, specimens, and GenBank accession numbers of sequences used in ITS+nLSU+EF1-α phylogenetic analyses.

Species	Specimen no.	GenBank accession no.			References
		ITS	nLSU	EF1-α	
*Fuscoporia acutimarginata*	Dai 15137	MH050751	MH050765	MN848821	Chen and Dai, 2019
*F. acutimarginata*	Dai 16892	MH050752	MH050766	MN848822	Chen and Dai, 2019
*F. ambigua*	Cui 9244	MN816706	MN809995	MN848804	Du et al., 2020
*F. ambigua*	JV 0509/151	MN816707	MN809996	−	Du et al., 2020
*F. americana*	JV 1209/3-J	−	MG008466	−	Chen et al., 2019
*F. americana*	JV 1209/100	KJ940022	MG008467	MH636384	Chen et al., 2019
*F. atlantica*	SP 445618	KP058515	KP058517	−	Pires et al., 2015
*F. atlantica*	SP 465829	KP058514	KP058516	−	Pires et al., 2015
*F. australasica*	Dai 15625	MN816726	MN810018	MN848829	Chen et al., 2020
*F. australasica*	Dai 15636	MG008397	MG008450	MH636408	Chen et al., 2019
*F. australiana*	Dai 18672	MN816703	MN810014	MN848848	Chen et al., 2020
*F. australiana*	Dai 18879	MN816705	MN810015	MN848850	Chen et al., 2020
*F. bambusae*	Dai 16599	MN816711	MN809999	MN848808	Chen et al., 2020
*F. bambusae*	Dai 16615	MN816715	MN810001	MN848810	Chen et al., 2020
*F. callimorpha*	Dai 17388	MN121765	MN121824	−	Chen and Dai, 2019
*F. callimorpha*	Doll 868	MN816701	MN809992	MN848840	Chen et al., 2020
*F. caymanensis*	JV 1908/74	MT676832	MT676833	−	Vlasák et al., 2020
*F. caymanensis*	JV 1408/5	MW009110	MW009109	−	Vlasák et al., 2020
*F. centroamericana*	JV 1607/93	MG008444	MG008460	MH636389	Chen et al., 2019
*F. centroamericana*	O 908267	MG008443	−	−	Chen et al., 2019
*F. chinensis*	Dai 15713	MN816721	MN810008	MN848846	Chen et al., 2020
*F. chinensis*	Cui 11209	MN121767	MN121826	−	Chen and Dai, 2019
*F. chrysea*	JV 1607/106-J	MN816736	MN810027	MN848818	Chen et al., 2020
*F. cinchonensis*	Dai 19815	**OP603023**	**OP600561**	−	Present study
*F. contigua*	Dai 16025	MG008401	MG008454	MH636386	Chen et al., 2019
*F. contigua*	Dai 13567A	MG008402	MG008455	MN848817	Chen and Dai, 2019
*F. costaricana*	JV 1407/92	MG008446	MG008461	MH636400	Chen et al., 2019
*F. costaricana*	JV 1504/85	MG008413	MG478454	MH636401	Chen et al., 2019
*F. dhofarensis*	ATN-007	OP593104	OP593105	OP597768	Hussain et al., 2022
*F. eucalypti*	Dai 18783	MN816730	MN810021	MN848832	Chen et al., 2020
*F. eucalypti*	Dai 18792	MN816731	MN810022	MN848831	Chen et al., 2020
*F. ferrea*	MUCL 45984	KX961112	KY189112	MH636403	Chen and Yuan, 2017
*F. ferrea*	Cui 11801	KX961101	KY189101	MN848823	Chen and Yuan, 2017
*F. ferruginosa*	JV 0408/28	KX961103	KY189103	MH636397	Chen and Yuan, 2017
*F. ferruginosa*	Dai 13200	MN816702	MN809993	MN848802	Chen et al., 2020
*F. gilva*	JV 0709/75 USA	MN816720	MN810007	MN848852	Chen et al., 2020
*F. gilva*	JV 1209/65	MN816719	MN810006	MN848851	Chen et al., 2020
‘*F. gilva*’	URM 83957	MH392545	MH407344	−	Yuan et al., 2020
‘*F. gilva*’	URM 91223	MH392550	MH407349	−	Yuan et al., 2020
*F. hainanensis*	Dai 16105	−	ON520809	ON616518	Chen et al., 2022
*F. hainanensis*	Dai 16110	−	ON520810	ON616519	Chen et al., 2022
*F. hawaiiana*	JV 2208/H22-J	OQ817709	OQ817855	OQ849746	Present study
*F. hawaiiana*	JV 2208/H30-J	OQ817710	OQ817856	OQ849747	Present study
*F. insolita*	Spirin 5251	KJ677113	−	−	Spirin et al., 2014
*F. insolita*	Spirin 5208	MN816724	MN810016	MN848800	Chen et al., 2020
*F. karsteniana*	Dai 16552	MN816716	MN810002	MN848806	Chen et al., 2020
*F. karsteniana*	Dai 11403	MN816717	MN810003	MN848807	Chen et al., 2020
*F. latispora*	JV 1109/48	MG008439	MG008468	MH636395	Chen et al., 2019
*F. latispora*	JV 0610/VII-Kout	MG008436	MG008469	MH636396	Chen et al., 2019
*F. licnoides*	URM 84107	MH392556	MH407355	−	Yuan et al., 2020
*F. licnoides*	URM 83001	MH392561	MH407357	−	Yuan et al., 2020
*F. marquesiana*	URM83094	MH392544	MH407343	−	Yuan et al., 2020
*F. minutissima*	JV 2208/H12-J	OQ817711	OQ817857	OQ849748	Present study
*F. minutissima*	JV 2208/H16-J	OQ817712	OQ817858	OQ849749	Present study
*F. monticola*	Dai 10909	MG008410	−	−	Chen et al., 2019
*F. monticola*	Dai 11860	MG008406	MG008457	MH636390	Chen et al., 2019
*F. palomari*	JV 1004/5-J	MN816737	−	−	Chen et al., 2020
*F. palomari*	JV 1305/3-J	MN816738	MN810028	MN848801	Chen et al., 2020
*F. plumeriae*	Dai 17814	MN816714	MN810011	MN848845	Chen et al., 2020
*F. plumeriae*	Dai 18858	MN816712	MN810010	MN848843	Chen et al., 2020
*F. pulviniformis*	CMW 48060	MH599101	MH599125	MT108959	Tchoumi et al., 2020
*F. pulviniformis*	CMW 48600	MH599102	MH599127	MT108960	Tchoumi et al., 2020
*F. punctatiformis*	Dai 17443	MH050755	MH050764	−	Chen and Dai, 2019
*F. punctatiformis*	Doll#872a	MH050753	−	−	Chen and Dai, 2019
*F. ramulicola*	Dai 15723	MH050749	MH050762	MN848824	Chen and Dai, 2019
*F. ramulicola*	Dai 16155	MH050750	MH050763	MN848825	Chen and Dai, 2019
*F. roseocinerea*	JV 1407/84	MN816740	MN810030	MN848819	Chen et al., 2020
*F. roseocinerea*	JV 1109/78-J	MN816742	MN810032	MN848820	Chen et al., 2020
*F. rufitincta*	JV 1008/25	KJ940029	KX058575	−	Chen et al., 2020
*F. rufitincta*	JV 0904/142	KJ940030	KX058574	−	Chen et al., 2019
*F. sarcites*	JV 0402/20K	MZ264225	MZ264218	−	Wu et al., 2022a
*F. scruposa*	CMW 47749	MH599106	MH599129	MT108963	Yuan et al., 2020
*F. scruposa*	CMW 48145	MH599105	MH599130	MT108962	Yuan et al., 2020
*F. semiarida*	URM83800	MH392562	MH407361	−	Yuan et al., 2020
*F. semiarida*	URM82510	MH392563	MH407362	−	Yuan et al., 2020
*F. senex*	MEL 2382630	KP012992	KP012992	−	Chen et al., 2020
*F. senex*	KAUNP MK41	KP794600	−	−	Chen et al., 2020
*F. septiseta*	Dai 12820	MG008405	MN810033	MH636394	Chen et al., 2019
*F. septiseta*	JV 0509/78	MG008404	−	−	Chen et al., 2019
*F. setifera*	Dai 15710	MH050758	MH050767	MN848841	Chen and Dai, 2019
*F. setifera*	Dai 15706	MH050759	MH050769	MN848842	Chen and Dai, 2019
*F. shoreae*	Dai 17806	MN816734	MN810025	MN848815	Chen et al., 2020
*F. shoreae*	Dai 17818	MN816735	MN810026	MN848816	Chen et al., 2020
*F. sinica*	Dai 15468	MG008412	MG008459	MH636392	Chen et al., 2019
*F. sinica*	Dai 15489	MG008407	MG008458	MH636393	Chen et al., 2019
*F. sinuosa*	Dai 20498	MZ264226	MZ264219	−	Wu et al., 2022a
*F. sinuosa*	Dai 20499	MZ264227	MZ264220	−	Wu et al., 2022a
*F. subchrysea*	Dai 16201	MN816708	MN809997	MN848811	Chen et al., 2020
*F. subchrysea*	Dai 17656	MN816709	MN809998	MN848812	Chen et al., 2020
*F. subferrea*	Dai 16326	KX961097	KY053472	MN848826	Chen and Yuan, 2017
*F. subferrea*	Dai 16327	KX961098	KY053473	−	Chen and Yuan, 2017
*F. submurina*	Dai 19501	MZ264229	MZ264222	−	Wu et al., 2022b
*F. submurina*	Dai 19655	MZ264228	MZ264221	−	Wu et al., 2022b
*F. torulosa*	JV 1405/2	KX961106	KY189106	MH636405	Chen and Yuan, 2017
*F. torulosa*	Dai 15518	MN816732	MN810023	MN848827	Chen et al., 2020
*F. viticola*	JV 0911/6	KX961110	−	−	Chen and Yuan, 2017
*F. viticola*	He 2123	MN816725	MN810017	−	Chen et al., 2020
*F. wahlbergii*	JV 1312/20-Kout	MN816727	MG008462	−	Chen et al., 2020
*F. wahlbergii*	JV 0709/169-J	MN816728	−	−	Chen et al., 2020
*F. yunnanensis*	Cui 8182	MH050756	MN810029	−	Chen and Dai, 2019
*F. yunnanensis*	Dai 15637	MH050757	MH050768	−	Chen and Dai, 2019
Outgroups					
*Coniferiporia weirii*	CFS 504	AY829341	AY829345	−	Zhou et al., 2016
*Phellinidium fragrans*	CBS 202.90	AY558619	AY059027	−	Zhou et al., 2016

Newly generated sequences for this study and new species are in bold.

### 2.3 Phylogenetic analysis

The following softwares were used for data processing and phylogenetic analyses: Mesquite, MAFFT 7.110, BioEdit 7.0.1 (Hall, 1999), ClustalX 2.0P10 (Thompson et al., 1997), MrModeltest 2.3 (Posada and Crandall, 1998; Nylander, 2004), PAUP* 4.0b10 (MP, Swofford, 2002), raxmlGUI 1.2 (ML, Stamatakis, 2006; Silvestro and Michalak, 2012), TreeView 1.5.0, and PowerPoint. Maximum parsimony (MP) and maximum likelihood (ML) methods were adopted to perform phylogenetic analyses of the two aligned datasets. The two phylogenetic methods resulted in similar topology for each data set. Thus, only the topology of the MP analysis appears along and branches that received bootstrap supports greater than or equal to 75% (MP and ML) were considered as significantly supported at the nodes.

**Figure 1:**
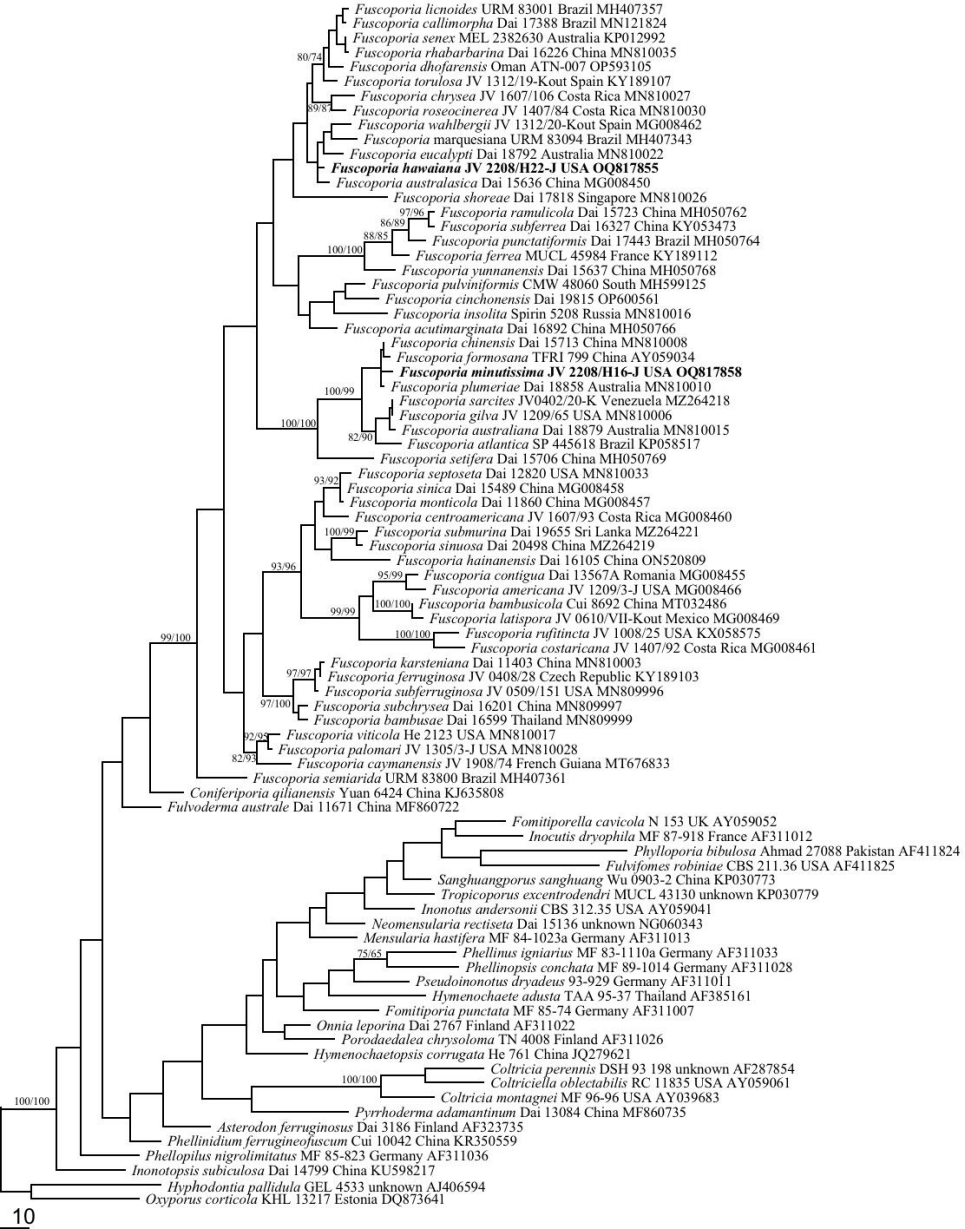
Phylogeny of *Fuscoporia* and related species generated inferred from nLSU sequences. Statistical values (MP/ML) are indicated for each node. Names of new species are in bold.

To explore the phylogenetic position of *Fuscoporia* in Hymenochaetaceae, representatives of 28 genera of Hymenochaetaceae were included in nLSU dataset (not shown in **Table 1** except for *Fuscoporia*; shown in **Figure 1**). *Oxyporus populinus* (Schumach.) Donk and *Hyphodontia pallidula* (Bres.) J. Erikss. were used as outgroups (Larsson et al., 2006; Zmitrovich and Malysheva, 2014; Zhou et al., 2016; Chen et al., 2019).

**Figure 2:**
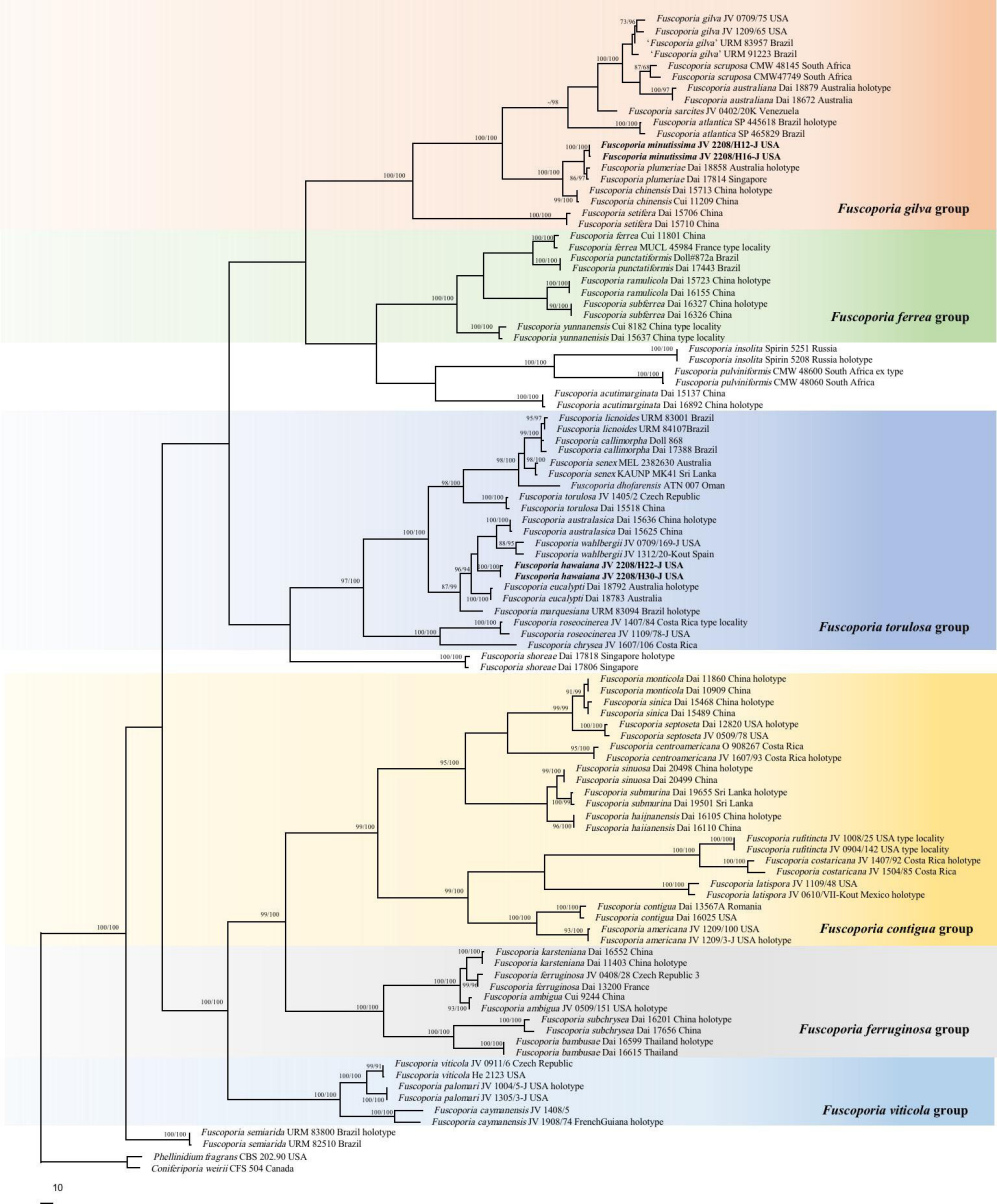
Phylogeny of *Fuscoporia* and related species generated inferred from ITS+nLSU+EF1-α dataset. Statistical values (MP/ML) are indicated for each node. Names of new species are in bold.

In addition to the newly generated sequences, additional ITS, nLSU, and EF1-α sequences of *Fuscoporia* based on formerly studies (Chen et al., 2019; Chen et al., 2020; Du et al., 2020; Wu et al., 2022a) were obtained from GenBank (**Table 1**) to explore the phylogenetic position of our specimens in *Fuscoporia*. A total of 251 sequences, 100 samples of *Fuscoporia* were analyzed in the ITS+nLSU+EF1-α dataset (**Figure 2**). *Coniferiporia weirii* (Murrill) L.W. Zhou et al (2016) and *Phellinidium fragrans* (M.J. Larsen & Lombard) Nuss (1986) were selected as outgroups (Zhou et al., 2016; Chen and Yuan, 2017).

## 3 Results

### 3.1 Phylogeny

To explore the phylogenetic position of *Fuscoporia* in Hymenochaetaceae, 81 taxa of the family Hymenochaetaceae were included in nLSU-alone dataset (**Figure 1**). The dataset had an aligned length of 1436 characters, of which 1019 were constant, 98 variable but parsimony-uninformative, and 291 parsimony-informative. Maximum parsimony analysis yielded 140 equally topologies (TL = 1723, CI = 0.327, RI = 0.672, RC = 0.220, HI = 0.673). *Fuscoporia* is a powerfully supported lineage (100/100) within the Hymenochaetaceae family based on phylogenetic tree inferred from the nLSU dataset (**Figure 1**).

The ITS+nLSU+EF1-α dataset (**Figure 2**) included 98 ITS, 92 nLSU, and 62 EF1-α sequences from 100 fungal specimens representing 52 pecies of *Fuscoporia*. The dataset had an aligned length of 2705 characters, of which 1721 were constant, 99 variable but parsimony-uninformative, and 885 parsimony-informative. Maximum parsimony analysis yielded six equally topologies (TL = 4489, CI = 0.389, RI = 0.807, RC = 0.314, HI = 0.611). Two well-supported lineages (100/100) clustered within *Fuscoporia* and distincted from other species in phylogeny (**Figure 2**). Taking morphological characters into consideration, these two new lineages represent two new species, *F. hawaiiana* and *F. minutissima*, which are described below.

### 3.2 Taxonomy

**Figure 3:**
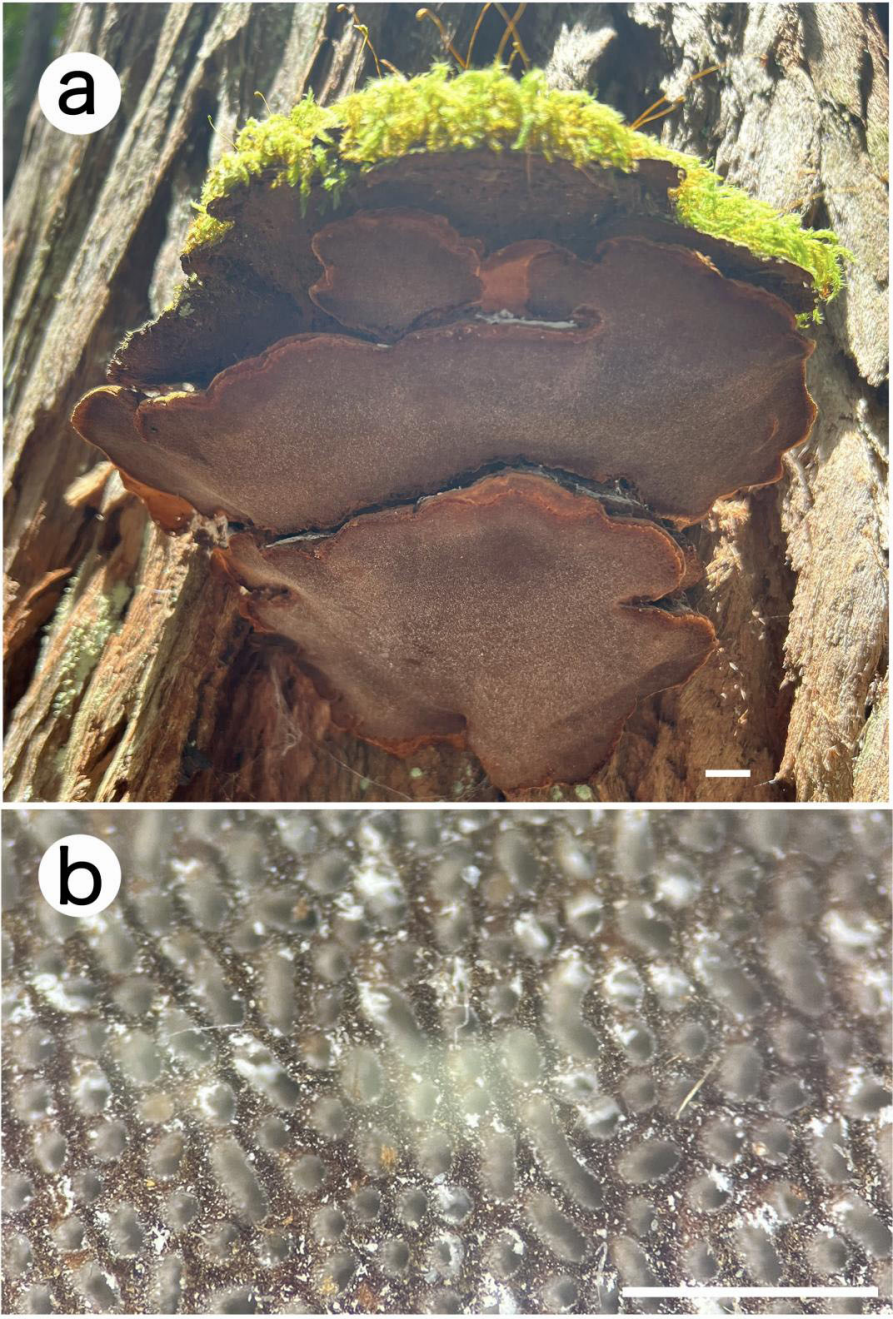
Basidiocarps of *Fuscoporia hawaiiana* (holotype, JV 2208/H22-J). Bars: **(A)** = 1 cm. **(B)** = 1 mm.

**Figure 4:**
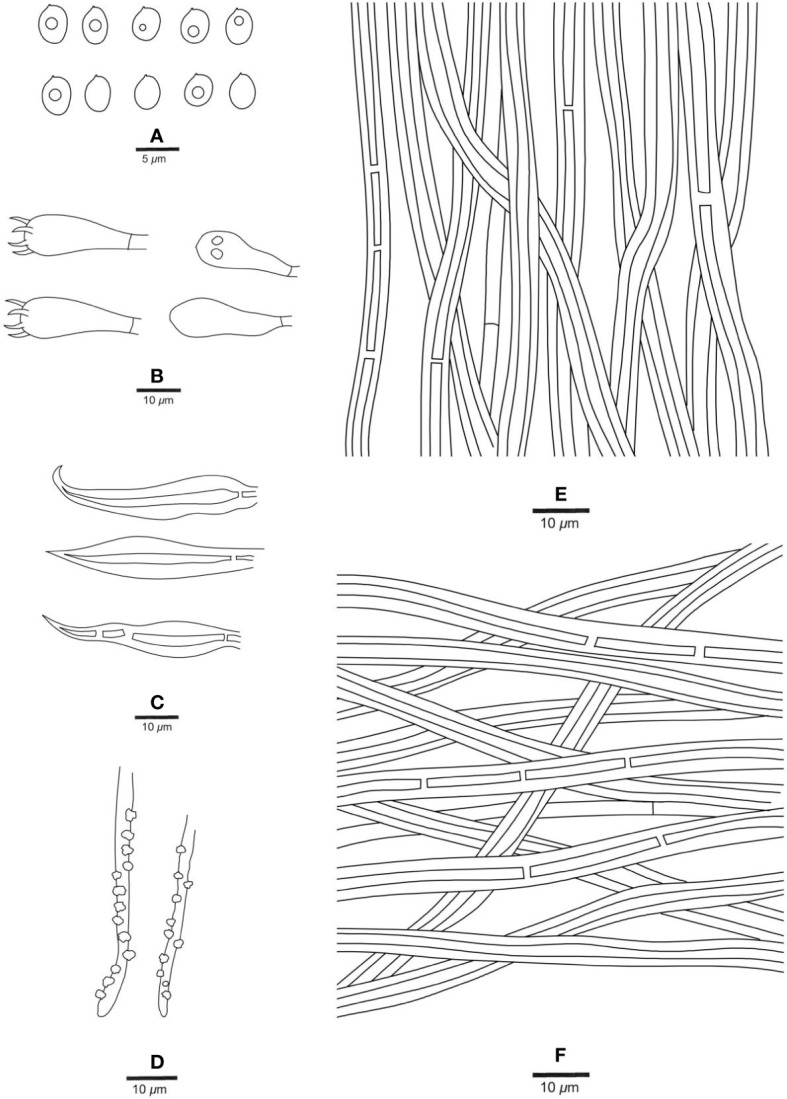
Microscopic structures of *Fuscoporia hawaiiana* (holotype, JV 2208/H22-J). **(A)** Basidiospores. **(B)** Basidia and basidioles. **(C)** Hymenial setae. **(D)** Generative hyphae at dissepiment edge. **(E)** Hyphae from tube trama. **(F)** Hyphae from subiculum.

*Fuscoporia hawaiiana* Q. Chen, Jing Si & Vlasák, sp. nov., **Figures 3**, **4**

MycoBank no. — MB 847960

*Holotype.* — USA. Hawaii, Big Island, Kalopa State Park, on living tree of *Eucalyptus*, August 2022, JV 2208/H22-J (PRM, isotype JV, BJFC 039915).

*Etymology.* — *Hawaiiana* (Lat.): refers to the place (Hawaii) where the species was collected.

*Fruiting body*. — Basidiocarps perennial, pileate, laterally fused to imbricate, without odor or taste when fresh, hard corky when dry. Pilei mostly semicircular, projecting up to 11 cm long, 5 cm wide, and 5 cm thick at the base, more or less convex towards margin. Pileal surface reddish brown, concentrically sulcate with zones, glabrous, sometimes covered with mosses; margin obtuse, yellowish brown, up to 5 mm wide. Pore surface honey-yellow to deep olive, slight glancing; margin narrow, olivaceous buff, up to 1 mm wide; pores circular, 5−7 per mm; dissepiments thin to fairly thick, entire, abundant hymenial setae in tube cavities (under anatomical lens). Context clay-buff, hard corky, about 8 mm thick. Tubes olivaceous buff, hard corky, up to 2 cm long.

*Hyphal structure*. — Hyphal system dimitic; generative hyphae simple septate; tissue darkening but otherwise unchanged in KOH.

*Context*. — Generative hyphae rare, hyaline, thin- to slightly thick-walled, branched, frequently simple septate, 2−2.5 μm in diam; skeletal hyphae dominant, rust-brown, thick-walled with a medium to wide lumen, unbranched, occasionally septate, straight, more or less straight and regularly arranged, 2.5−3.5 μm in diam.

*Tubes*. — Generative hyphae rare, mostly present at dissepiment edges and subhymenium, hyaline, thin-walled, frequently branched and simple septate, 1.5−2.5 μm in diam, some of them encrusted at dissepiment edges and in the hymenium; skeletal hyphae dominant, yellowish brown, thick-walled with a medium to wide lumen, frequently septate, more or less straight, subparallel along the tubes, 2.5−3.5 μm in diam. Hymenial setae subulate, occasionally hooked, mostly originating from tramal hyphae, dark brown, thick-walled, 30−45 × 4−7 μm; basidia broadly clavate, with four sterigmata and a simple septum at the base, 12−18 × 5−7 μm; basidioles dominating the hymenium, in shape similar to basidia, but slightly smaller.

*Spores*. — Basidiospores broadly ellipsoid, hyaline, thin-walled, smooth, IKI−, CB−, some of them bearing a guttule, 4−6 × (3.4−)3.5−4.5(−4.7) μm, L = 4.77 μm, W = 3.90 μm, Q = 1.14−1.31 (n = 60/2).

*Other material examined (paratype)*. — USA. Hawaii, Big Island, Volcano, on dead tree of *Metrosideros polymorpha*, August 2022, JV 2208/H30-J (JV, BJFC 039918).

**Figure 5:**
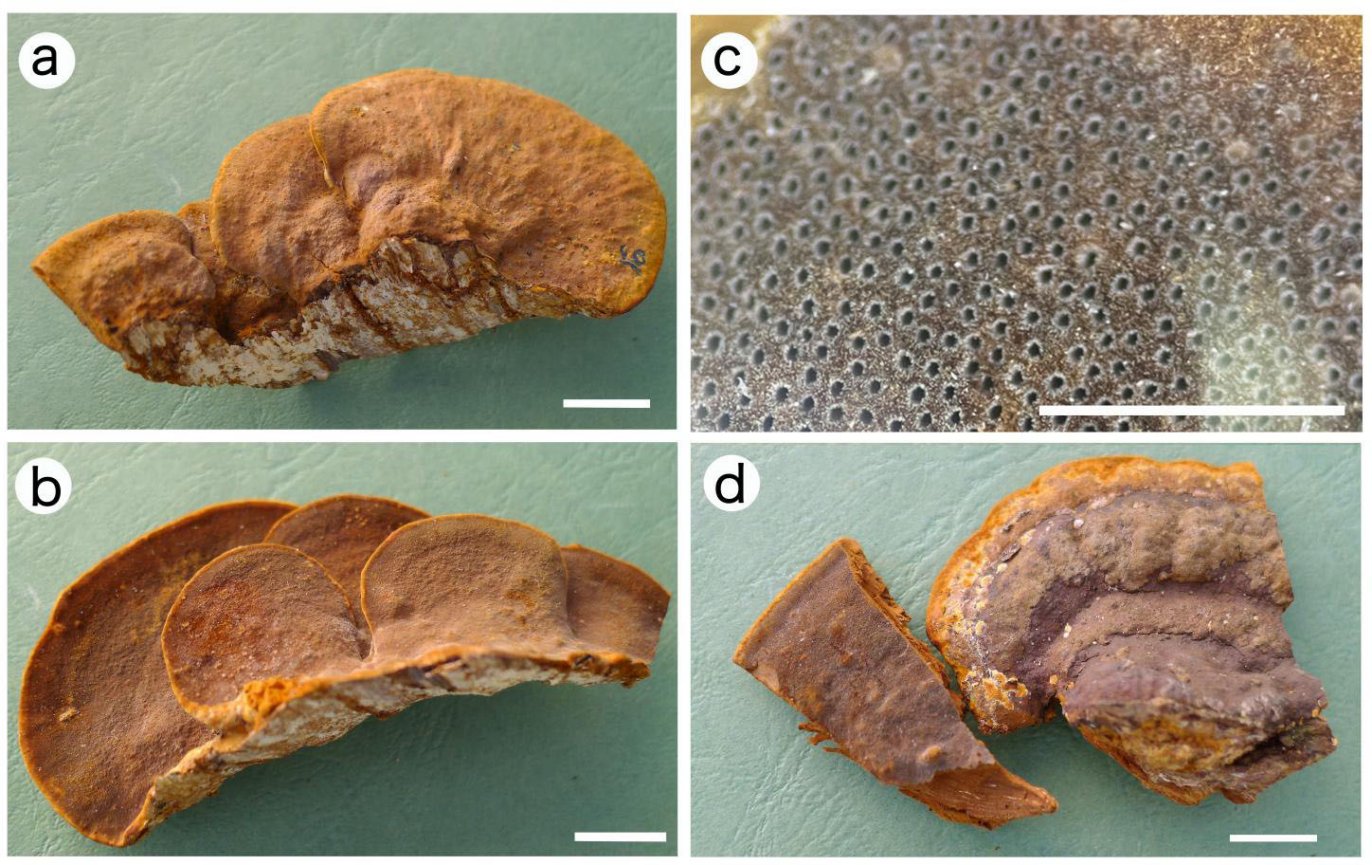
Basidiocarps of *Fuscoporia minutissima*. **(A–C)**JV 2208/H16-J (holotype). **(D)** JV 2208/H12-J. Bars: **(A, B, D)** = 1 cm. **(C)** = 1 mm.

**Figure 6:**
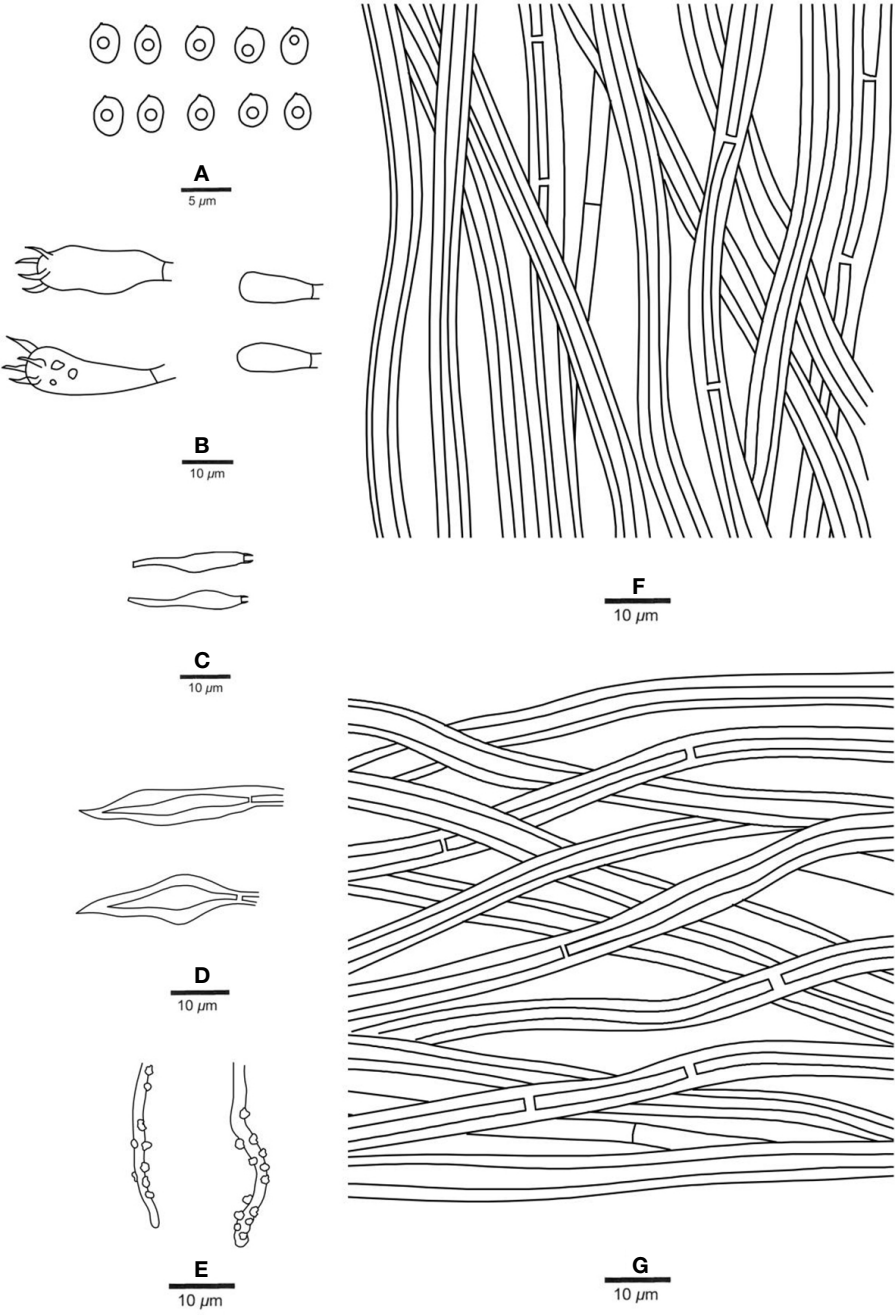
Microscopic structures of *Fuscoporia minutissima* (holotype, JV 2208/H16-J). **(A)** Basidiospores. **(B)** Basidia and basidioles. **(C)** Cystidioles. **(D)** Hymenial setae. **(E)** Generative hyphae at dissepiment edge. **(F)** Hyphae from tube trama. **(G)** Hyphae from subiculum.

*Fuscoporia minutissima* Q. Chen, Jing Si & Vlasák, sp. nov., **Figures 5**, **6** MycoBank no. — MB 847964

*Holotype*. — USA. Hawaii, Big Island, Makuala O’oma Trail, August 2022, JV 2208/H16-J (PRM, isotype JV, BJFC 039911).

*Etymology*. — *Minutissima* (Lat.): refers to the very small size of the pores exhibiting by these species.

*Fruiting body*. — Basidiocarps perennial, pileate, imbricate, without odor or taste when fresh, hard corky when dry. Pilei mostly imbricate, projecting up to 5 cm long, 3 cm wide, and 1 cm thick at the base. Pileal surface reddish brown, concentrically sulcate with zones, nodulose; margin obtuse to slightly acute, honey-yellow, up to 1 mm wide. Pore surface greyish brown to deep olive, glancing; margin narrow, honey-yellow, up to 1 mm wide; pores circular, 10−13 per mm; dissepiments fairly thick, entire and matted, abundant hymenial setae in tube cavities (under anatomical lens). Context honey-yellow, hard corky, up to 5 mm thick. Tubes olivaceous buff, paler than pores, hard corky, up to 4 mm long.

*Hyphal structure*. — Hyphal system dimitic; generative hyphae simple septate; tissue darkening but otherwise unchanged in KOH.

*Context*. — Generative hyphae rare, hyaline, thin- to slightly thick-walled, unbranched, frequently simple septate, 2−3 μm in diam; skeletal hyphae dominant, rust-brown, thick-walled with a medium to narrow lumen, unbranched, occasionally septate, straight, regularly arranged, 3−4 μm in diam.

*Tubes*. — Generative hyphae rare, mostly present at dissepiment edges and subhymenium, hyaline, thin-walled, frequently branched and simple septate, 2−3 μm in diam, some of them encrusted at dissepiment edges and in the hymenium; skeletal hyphae dominant, yellowish brown, thick-walled with a medium to narrow lumen, frequently septate, more or less straight, subparallel along the tubes, 3−4 μm in diam. Hymenial setae subulate, mostly originating from tramal hyphae, dark brown, thick-walled, 18−40 × 6−9 μm; fusoid cystidioles hyaline and thin-walled, 9.5−12 × 4−5.5 μm; basidia short clavate to barrel-shaped, with four sterigmata and a simple septum at the base, 10−15 × 4−6.5 μm; basidioles dominating the hymenium, in shape similar to basidia, but slightly smaller.

*Spores*. — Basidiospores broadly ellipsoid to subglobose, hyaline, thin-walled, smooth, IKI−, CB−, bearing a guttule, (3−)3.4−4 × (2.2−)2.4−3(−3.8) μm, L = 3.60 μm, W = 2.79 μm, Q = 1.24−1.30 (n = 52/2).

*Other material examined (paratype)*. — USA. Hawaii, Kauai Island, Koke’e State Park, on dead tree of *Acacia koa*, August 2022, JV 2208/H12-J (JV, BJFC 039910).

## 4 Discussion

The islands far from the mainland have attracted scientific researchers for a long time, due to the close combination of ecological environment and evolution process here that deepen our understanding of the formation process of biological diversity (Cotoras et al., 2018). Hawaii is a biological hotspot with a variety of climates and habitats. 160 genera and 400 species of wood-rotting basidiomycetes are reported from 110 native and exotic substrate species on the Hawaiian Islands (Gilbertson et al., 2002; Ashiglar et al., 2015). There are few natural forests on the Hawaii islands (Chambers et al., 2007). Most forests are filled with introduced trees, and our new species may be introduced as well. The two new wood-rotting fungal species *Fuscoporia hawaiiana* and *F. minutissima* were collected from the northernmost and oldest Kauai Island, and the largest island Big Island in the Hawaiian Islands, both with a tropical sea climate.

*Fuscoporia hawaiiana* is characterized by perennial and pileate basidiocarps, circular and medium pores (5−7 per mm), the absence of cystidioles, hooked hymenial setae, broadly ellipsoid to subglobose basidiospores measuring 4−6 × 3.5−4.5 μm. *Fuscoporia hawaiiana* may also be distributed in Yunnan Province, China, considered to be *F. torulosa* derived from CLZhao 10146 (OM959398) on GenBank. The ITS of CLZhao 10146 shows that only 2 base differences from our new samples, but unfortunately, it lacks nLSU or EF1-α sequence. Furthermore, *F. torulosa* can be easily distinguished from *F. hawaiiana* by its subungulate basidiocarps and straight hymenial setae. Morphologically, seven species, *F. semihispida* (Ryvarden) Y.C. Dai & F. Wu, *F. australasica* Q. Chen, F. Wu & Y.C. Dai, *F. marquesiana* Gibertoni & C.R.S. de Lira, *F. atlantica* Motato-Vásq., R.M. Pires & Gugliotta, *F. wahlbergii* (Fr.) T. Wagner & M. Fisch., *F. eucalypti* Q. Chen, F. Wu & Y.C. Dai, and *F. rufa* (Bres.) Y.C. Dai & F. Wu., are similar to *F. hawaiiana* by sharing similar pileate basidiocarps and hooked hymenial setae. However, *F. hawaiiana* is distant from *F. atlantica*, *F. marquesiana*, *F. wahlbergii*, *F. eucalypti*, and *F. australasica* both in the phylogenetic analyses (**Figures 1**, **2**) and morphology. *F. atlantica* differs from *F. hawaiiana* by its annual basidiocarps (Pires et al., 2015); *F. australasica* is described from Southern China and Viet Nam and differs from *F. hawaiiana* by its glabrous basidiocarps and the presence of cystidioles (Chen et al., 2020); *F. wahlbergii* is described from Europe, East-African, Australia, and USA, and distinguishes from *F. hawaiiana* by its smaller pores (7−9 per mm) and the presence of cystidioles (Chen et al., 2020); *F. eucalypti* is distributed Australia, and differs from *F. hawaiiana* by its spores effused-reflexed to pileate and wider (4.3−5.5 × 4−4.5 μm) (Chen et al., 2020); *F. marquesiana* is a South American species, and can be easily distinguished from *F. hawaiiana* by its smaller pores (8−9 per mm) (Wu et al., 2022b). Even without molecular data, *F. semihispida* and *F. rufa* can be easily distinguished from *F. hawaiiana* by narrower or wider spores (2−2.5 μm in *F. semihispida*; 4−4.5 μm in *F. rufa*) (Wu et al., 2022a).

*Fuscoporia minutissima* stands out in the genus by its perennial and pileate basidiocarps, and very small pores (10−13 per mm) and spores (3.4−4 × 2.4−3 μm). *Fuscoporia minutissima* is usually sterile after collecting, and such specimens are very similar to *F. gilva* by pileate basidiocarps, glabrous to rugose sometimes with nodulose pileal surface and broadly ellipsoid to subglobose spores, but the later differs by having bigger pores (6−8 per mm). *Fuscoporia plumeriae* Q. Chen, F. Wu & Y.C. Dai and *F. chinensis* Q. Chen, F. Wu & Y.C. Dai clustered together with *F. minutissima* into a group with a strong support (100/100, **Figures 1**, **2**). Morphologically, these species share the effused-reflexed to pileate basidiocarps with lacerate dissepiments and ellipsoid basidiospores; but *F. plumeriae* and *F. chinensis* have annual basidiocarps and bigger pores (10−13 per mm in *F. minutissima*; 8−10 per mm in *F. plumeriae*; 7−8 per mm in *F. chinensis*; Chen et al., 2020). *Fuscoporia rhabarbarina* (Berk.) Groposo, Log.-Leite & Góes-Neto is a common fungus on hardwoods along streams in South America and subtropical and tropical Asia. But the type locality of this species remains unknown, probably in Brazil (Wu et al., 2022b). *Fuscoporia minutissima* may be confused with *F. rhabarbarina* in field, but the pileal surface of *F. rhabarbarina* is glabrous and dark brown crust at the base. Moreover, the two species are not closely related in the phylogenetic tree (**Figure 1**).

*Fuscoporia hawaiiana* and *F. minutissima* are described in this study. Traditionally, they were most probably treated as *F. wahlbergii* and *F. gilva*, respectively. The phylogenetic analyses provide molecular evidence to support these two new species derive from the *F. wahlbergii* complex and the *F. gilva* complex (**Figures 1**, **2**). The similar results are demonstrated in many polypores complex, for instances, *Megasporoporia setulosa* (Henn.) Rajchenb. (Wang et al., 2021; Wang et al., 2022), *Heterobasidion annosum* (Fr.) Bref. (Yuan et al., 2021), *Porogramme epimiltina* (Berk. & Broome) Y.C. Dai (Mao et al., 2023), *Sidera vulgaris* (Fr.) Miettinen (Liu et al., 2021), and *Phaeolus schweinitzii* (Fr.) Pat. (Yuan et al., 2022). It seems that the diversity of wood-inhabiting fungi is extremely rich in tropics, some traditional definition on tropical species should be re-evaluated, and the concepts of taxa in tropics should be modified after molecular phylogeny.

**A key to North American species of *Fuscoporia.***

1. Basidiocarps completely resupinate...........................................1
- Basidiocarps pileate, effused-reflexed or substipitate................7
2. Mycelial setae absent .........................*F. ferrea* (Pers.) G. Cunn.
- Mycelial setae present.....................................................................3
3. Pores 5−9 per mm..........................................................................4
- Pores 2−4 per mm...........................................................................5
4. Pores 7−9 per mm, skeletal hyphae septate ................ ............................................................................................................ ......*F. rufitincta* (Berk. & M.A. Curtis ex A.L. Sm.) Murrill
- Pores 5−6 per mm, skeletal hyphae aseptate *F. ambigua* P. Du, Q. Chen & J. Vlasák
5. Basidiocarps perennial ..........................*F. contigua* (Pers.) G. Cunn.
- Basidiocarps annual.................................................................................6
6. Spores broadly ellipsoid, 4.8−6.0 × 3.2−4.2 μm ....................................*F. americana* Y.C. Dai, Q. Chen & J. Vlasák
- Spores cylindric, 6.0−7.0 × 2.0−3.0 μm ............................*F. septiseta* Y.C. Dai, Q. Chen & J. Vlasák
7. Hymenial setae hooked ........................................................................8
- Hymenial setae straight...........................................................................9
8. Pores 7−9 per mm, cystidioles present..........................*F. wahlbergii*
- Pores 5−7 per mm, cystidioles absent.............................*F. hawaiiana*
9. Basidiocarps annual.........................*F. palomari* Vlasák & Ryvarden
- Basidiocarps perennial..........................................................................10
10. Spores cylindric, 7−9 × 1.5−2 μm, Q > 2...........................*F. viticola* (Schwein.) Murrill
- Spores broadly ellipsoid to ellipsoid, Q < 2.......................................11
11. Pileal surface velutinate to glabrous.......................*F. rhabarbarina*
- Pileal surface tomentose, hispid, rugose............................................12
12. Pores 10−13 per mm...................................................*F. minutissima*
- Pores 6−9 per mm..................................................................................13
13. Pileal surface not sulcate...........................................................*F. gilva*
- Pileal surface concentrically sulcate....................................................14
14. Basidiocarps subungulate, pileal surface grayish brown.....................................................................................*F. torulosa*
- Basidiocarps usually applanate, pileal surface fuscous to black........................................*F. senex* (Nees & Mont.) Ghob.-Nejh

## Data availability statement

The datasets presented in this study can be found in online repositories. The names of the repository/repositories and accession number(s) can be found below: *F. hawaiiana* JV 2208/H22-J: OQ817709; OQ817855; OQ849746. *F. hawaiiana* JV 2208/H30-J: OQ817710; OQ817856; OQ849747. *F. minutissima* JV 2208/H12-J: OQ817711; OQ817857; OQ849748. *F. minutissima* JV 2208/H16-J: OQ817712; OQ817858; OQ849749. *F. cinchonensis* OP603023; OP600561.

## Author contributions

QC, LL, JS, and JV designed the research and contributed to data analysis and interpretation. JV prepared the samples. QC and LL conducted the molecular experiments and analyzed the data. QC prepared the samples and drafted the manuscript. JV and JS discussed the results and edited the manuscript. All authors contributed to the article and approved the submitted version.

## References

[B1] Anonymous (1969). Flora of British fungi. colour identification chart (Edinburgh, UK: Her Majesty’s Stationery Office).

[B2] Ashiglar S. M. Brooks F. Cannon P. G. Klopfenstein N. B. (2015). “Preliminary survey of wood-associated fungi in southeast o’ahu of HawaiI using DNA-based identification,” in Proceedings of the 62nd annual Western international forest disease work conference. Eds. Murray M. Palacios P. (Cedar City, Utah, US), 67–69.

[B3] Chambers J. Q. Asner G. P. Morton D. C. Anderson L. O. Saatchi S. S. Espírito-Santo F. D. B.. (2007). Regional ecosystem structure and function: ecological insights from remote sensing of tropical forests. Trends Ecol. Evol. 22, 414–423. doi: 10.1016/j.tree.2007.05.001 17493704

[B4] Chen Q. Dai Y. C. (2019). Two new species of *Fuscoporia (*Hymenochaetales, basidiomycota) from southern China based on morphological characters and molecular evidence. MycoKeys 61, 75–89. doi: 10.3897/mycokeys.61.46799 31871407 PMC6923280

[B5] Chen Q. Du P. Vlasak J. Wu F. Dai Y. C. (2020). Global diversity and phylogeny of *Fuscoporia* (Hymenochaetales, basidiomycota). Mycosphere 11, 1477–1513. doi: 10.5943/mycosphere/11/1/10

[B6] Chen Q. Liu L. Zhang D. S. Dong L. L. (2022). *Fuscoporia hainanensis* sp. nov. (Hymenochaetales, basidiomycota), a new member of the *F. contigua* group. Phytotaxa 558, 251–262. doi: 10.11646/phytotaxa.558.3.1

[B7] Chen Q. Wu F. Ji X. H. Si J. Zhou L. W. Tian X. M.. (2019). Phylogeny of the genus *Fuscoporia* and taxonomic assessment of the *F. contigua* group. Mycologia 111, 423–444. doi: 10.1080/00275514.2019.1570749 30964428

[B8] Chen Q. Yuan Y. (2017). A new species of *Fuscoporia* (Hymenochaetales, basidiomycota) from southern China. Mycosphere 8, 1238–1245. doi: 10.5943/mycosphere/8/6/9 PMC6923280 31871407

[B9] Cotoras D. D. Bi K. Brewer M. S. Lindberg D. R. Prost S. Gillespie R. G. (2018). Co-Occurrence of ecologically similar species of Hawaiian spiders reveals critical early phase of adaptive radiation. BMC Evol. Biol. 18, 100. doi: 10.1186/s12862-018-1209-y 29921226 PMC6009049

[B10] Dai Y. C. Yang Z. L. Cui B. K. Wu G. Yuan H. S. Zhou L. W.. (2021). Diversity and systematics of the important macrofungi in Chinese forests. Mycosystema 40, 770–805. doi: 10.13346/j.mycosystema.210036

[B11] Du P. Chen Q. Vlasák J. (2020). *Fuscoporia ambigua* sp. nov., a new species from America and China. Phytotaxa 456, 175–185. doi: 10.11646/phytotaxa.456.2.5

[B12] Fiasson J. L. Niemela T. (1984). The hymenochaetales: a revision of the European poroid taxa. Karstenia 24, 14–28. doi: 10.29203/ka.1984.224

[B13] Gilbertson R. L. Bigelow D. M. Hemmes D. E. Desjardin D. E. (2002). Annotated check list of wood-rotting basidiomycetes of Hawaii. Mycotaxon 82, 215–239.

[B14] Gilbertson R. L. Ryvarden L. (1987). North American polypores 2 (Oslo: Fungiflora).

[B15] Hall T. A. (1999). Bioedit: a user-friendly biological sequence alignment editor and analysis program for windows 95/98/NT. Nucleic Acids Symp. Ser. 41, 95–98. doi: 10.1021/bk-1999-0734.ch008

[B16] Hussain S. Al-Kharousi M. Al-Muharabi M. A. Al-Maqbali D. Al-Shabibi Z. Al-Balushi A. H.. (2022). Phylogeny, distribution and time divergence of *Fuscoporia* (Hymenochaetaceae, basidiomycota) with the description of a new species from dhofar region, southern part of Oman. Phytotaxa 570, 150–164. doi: 10.11646/phytotaxa.570.2.3

[B17] Larsen M. J. Cobb-Poulle L. A. (1990). *Phellinus* (Hymenochaetaceae). a survey of the world taxa. *Synop* . Fungorum. 3, 1–206. doi: 10.2307/3760175

[B18] Larsson K. H. Parmasto E. Fischer M. Langer E. Nakasone K. K. Redhead S. A. (2006). Hymenochaetales: a molecular phylogeny for the hymenochaetoid clade. Mycologia 98, 926–936. doi: 10.1080/15572536.2006.11832622 17486969

[B19] Liu Z. B. Wu Y. D. Zhao H. Lian Y. P. Wang Y. R. Wang C. G.. (2022). Outline, divergence times, and phylogenetic analyses of *Trechisporales* (Agaricomycetes, basidiomycota). Front. Microbiol. 13. doi: 10.3389/fmicb.2022.818358 PMC9083364 35547118

[B20] Liu Z. B. Zhou M. Yuan Y. Dai Y. C. (2021). Global diversity and taxonomy of *Sidera* (Hymenochaetales, basidiomycota): four new species and keys to species of the genus. J. Fungi 7, 251. doi: 10.3390/jof7040251 PMC8066320 33810364

[B21] Lowe J. L. (1966). Polyporaceae of north america. the genus poria Vol. 90 (New York, US: Technical Publication of the State University College of Forestry at Syracuse University), 1–183.

[B22] Mao W. L. Wu Y. D. Liu H. G. Yuan Y. Dai Y. C. (2023). A contribution to *Porogramme* (Polyporaceae, agaricomycetes) and related genera. IMA Fungus 14, 5. doi: 10.1186/s43008-023-00110-z 36882814 PMC9990255

[B23] Murrill W. A. (1907). (Agaricales) *Polyporaceae* . North Am. Flora 9, 1–131.

[B24] Nylander J. A. A. (2004). MrModeltest v2. uppsala: program distributed by the author (Sweden: Evolutionary Biology Centre, Uppsala University).

[B25] Overholts L. D. (1953). The polyporaceae of the united states, Alaska and Canada (Ann Arbor: University of Michigan Press). doi: 10.2307/2481836

[B26] Petersen J. H. (1996). The Danish mycological society’s colour-chart (Greve: Foreningen til Svampekundskabens Fremme), 1–6.

[B27] Pires R. M. Motato-Vásquez V. de Gugliotta A. M. (2015). *Fuscoporia* atlantica sp. nov., a new polypore from the Brazilian Atlantic rainforest. Mycotaxon 130, 843–855. doi: 10.5248/130.843

[B28] Posada D. Crandall K. A. (1998). Modeltest: testing the model of DNA substitution. Bioinformatics 14, 817–818. doi: 10.1093/bioinformatics/14.9.817 9918953

[B29] Rehner S. A. Buckley E. (2005). A *Beauveria* phylogeny inferred from nuclear ITS and EF1-α sequences: evidence for cryptic diversification and links to *Cordyceps* teleomorphs. Mycologia 97, 84–98. doi: 10.1080/15572536.2006.11832842 16389960

[B30] Ryvarden L. Johansen I. (1980). A preliminary polypore flora of East Africa (Oslo: Fungiflora). doi: 10.2307/3759822

[B31] Si J. Zhang Y. Z. Liang J. Q. Li H. J. (2023). Morphology and phylogeny identify two new species and one new subspecies of *Podoscypha* from yunnan province, southwest China. Front. Microbiol. 14. doi: 10.3389/fmicb.2023.1151365 PMC10011068 36925482

[B32] Silvestro D. Michalak I. (2012). raxmlGUI: a graphical front-end for rAxML. Org. Divers. Evol. 12, 335–337. doi: 10.1007/s13127-011-0056-0

[B33] Spirin V. Vlasák J. Niemelä T. (2014). *Fuscoporia insolita* (Hymenochaetales, basidiomycota), a new species from Russian far East. Ann. Bot. Fenn. 51, 403–406. doi: 10.5735/085.051.0607

[B34] Stamatakis A. (2006). rAxML-VI-HPC: maximum likelihood-based phylogenetic analyses with thousands of taxa and mixed models. Bioinformatics 22, 2688–2690. doi: 10.1093/bioinformatics/btl446 16928733

[B35] Swofford D. L. (2002). PAUP*: phylogenetic analysis using parsimony (*and other methods). version 4.0b10 (Sunderland, MA: Sinauer Associates). doi: 10.1002/0471650129.dob0522

[B36] Tchoumi J. M. T. Coetzee M. P. A. Rajchenberg M. Roux J. (2020). Poroid hymenochaetaceae associated with trees showing wood-rot symptoms in the garden route national park of south Africa. Mycologia 112, 722–741. doi: 10.1080/00275514.2020.1753160 32574523

[B37] Thompson J. D. Gibson T. J. Plewniak F. Jeanmougin F. Higgins D. G. (1997). The Clustal_X windows interface: flexible strategies for multiple sequence alignment aided by quality analysis tools. Nucleic Acids Res. 25, 4876–4882. doi: 10.1093/nar/25.24.4876 9396791 PMC147148

[B38] Vilgalys R. Hester M. (1990). Rapid genetic identification and mapping of enzymatically amplified ribosomal DNA from several *Cryptococcus* species. J. Bacteriol. 172, 4238–4246. doi: 10.1128/jb.172.8.4238-4246.1990 2376561 PMC213247

[B39] Vlasák J. Kout J. Chen Q. Dai Y. C. (2020). *Fuscoporia caymanensis* sp. nov. (Basidiomycota, hymenochaetaceae), a new species from tropical America. Phytotaxa 472, 135–146. doi: 10.11646/phytotaxa.472.2.4

[B40] Wagner T. Fischer M. (2001). Natural groups and a revised system for the European poroid hymenochaetales (Basidiomycota) supported by nLSU rDNA sequence data. Mycol. Res. 105, 773–782. doi: 10.1017/S0953756201004257

[B41] Wagner T. Fischer M. (2002). Proceedings towards a natural classification of the worldwide taxa *Phellinus* s.l. and *Inonotus* s.l., and phylogenetic relationships of allied genera. Mycologia 94, 998–1016. doi: 10.1080/15572536.2003.11833156 21156572

[B42] Wang Y. R. Dai Y. C. Liu H. G. Vlasák J. Buchanan P. Yuan Y.. (2022). A new contribution to *Megasporoporia* sensu lato: six new species and three new combinations. Front. Microbiol. 13. doi: 10.3389/fmicb.2022.1046777 PMC9777752 36569086

[B43] Wang Y. R. Wu Y. D. Vlasak J. Yuan Y. Dai Y. C. (2021). Phylogenetic analysis demonstrating four new species in *Megasporoporia* sensu lato (Polyporales, basidiomycota). Mycosphere 12, 1012–1037. doi: 10.5943/mycosphere/12/1/11

[B44] White T. J. Bruns T. D. Lee S. Taylor J. (1990). “Amplification and direct sequencing of fungal ribosomal RNA genes for phylogenetics,” in PCR protocols: a guide to methods and applications. Eds. Innis M. A. Gelfand D. H. Sninsky J. J. White T. J. (US: New York Academic Press), 315–322. doi: 10.1016/B978-0-12-372180-8.50042-1

[B45] Wu F. Man X. W. Tohtirjap A. Dai Y. C. (2022a). A comparison of polypore funga and species composition in forest ecosystems of China, north America, and Europe. For. Ecosyst. 9, 100051. doi: 10.1016/j.fecs.2022.100051

[B46] Wu F. Zhou L. W. Vlasák J. Dai Y. C. (2022b). Global diversity and systematics of hymenochaetaceae with poroid hymenophore. Fungal Divers. 113, 1–192. doi: 10.1007/s13225-021-00496-4

[B47] Wu F. Zhou L. W. Yang Z. L. Bau T. Li T. H. Dai Y. C. (2019). Resource diversity of Chinese macrofungi: edible, medicinal and poisonous species. Fungal Divers. 98, 1–76. doi: 10.1007/s13225-019-00432-7

[B48] Yuan Y. Chen J. J. Korhonen K. Martin F. Dai Y. C. (2021). An updated global species diversity and phylogeny in the forest pathogenic genus *Heterobasidion* (Basidiomycota, russulales). Front. Microbiol. 11. doi: 10.3389/fmicb.2020.596393 PMC7817714 33488542

[B49] Yuan H. S. Lu X. Dai Y. C. Hyde K. D. Kan Y. H. Kušan I.. (2020). Fungal diversity notes 1277–1386: taxonomic and phylogenetic contributions to fungal taxa. Fungal Divers. 104, 1–266. doi: 10.1007/s13225-020-00461-7

[B50] Yuan Y. Wu Y. D. Wang Y. R. Zhou M. Qiu J. Z. Li D. W.. (2022). Two new forest pathogens in *Phaeolus* (Polyporales, basidiomycota) on Chinese coniferous trees were confirmed by molecular phylogeny. Front. Microbiol. 13. doi: 10.3389/fmicb.2022.942603 PMC9532751 36212865

[B51] Zhang Q. Y. Liu Z. B. Liu H. G. Si J. (2023). Two new corticioid species of phanerochaetaceae (Polyporales, basidiomycota) from southwest China. Front. Cell. Infect. Microbiol. 13. doi: 10.3389/fcimb.2023.1105918 PMC9936140 36816592

[B52] Zhou H. M. Bau T. Si J. (2023). Morphological and phylogenetic evidence reveal three new *Pseudohydnum* (Auriculariales, basidiomycota) species from north China. Front. Cell. Infect. Microbiol. 13. doi: 10.3389/fcimb.2023.1139449 PMC9975552 36875530

[B53] Zhou L. W. Vlasák J. Dai Y. C. (2016). Taxonomy and phylogeny of *Phellinidium* (Hymenochaetales, basidiomycota): a redefinition and the segregation of *Coniferiporia* gen. nov. for forest pathogens. Fungal Biol. 120, 988–1001. doi: 10.1016/j.funbio.2016.04.008 27521630

[B54] Zmitrovich I. V. Malysheva V. F. (2014). Studies on *Oxyporus* i. segregation of *Emmia* and general topology of phylogenetic tree. Mycol. Phytopathol. 48, 161–171.

